# Microbial Community of Saline, Alkaline Lakes in the Nebraska Sandhills Based on 16S rRNA Gene Amplicon Sequence Data

**DOI:** 10.1128/MRA.00063-19

**Published:** 2019-03-21

**Authors:** Nicole A. Fiore, David D. Dunigan, Julie J. Shaffer, Ryan Roberts, Sanjay Antony-Babu, Bradley A. Plantz, Kenneth W. Nickerson, Andrew K. Benson, Karrie A. Weber

**Affiliations:** a School of Biological Sciences, University of Nebraska—Lincoln, Lincoln, Nebraska, USA; b Department of Plant Pathology, University of Nebraska—Lincoln, Lincoln, Nebraska, USA; c Nebraska Center for Virology, University of Nebraska—Lincoln, Lincoln, Nebraska, USA; d Department of Biology, University of Nebraska at Kearney, Kearney, Nebraska, USA; e Department of Biochemistry, University of Nebraska—Lincoln, Lincoln, Nebraska, USA; f Department of Food Science and Technology, University of Nebraska—Lincoln, Lincoln, Nebraska, USA; g Department of Earth and Atmospheric Sciences, University of Nebraska—Lincoln, Lincoln, Nebraska, USA; h Daughtery Water for Food Institute, University of Nebraska—Lincoln, Lincoln, Nebraska, USA; Broad Institute of MIT and Harvard University

## Abstract

The Nebraska Sandhills region contains over 1,500 geochemically diverse interdunal lakes, some of which are potassium rich, alkaline, and hypersaline. Here, we report 16S rRNA amplicon pyrosequencing data on the water and sediment microbial communities of eight alkaline lakes in the Sandhills of western Nebraska.

## ANNOUNCEMENT

The Nebraska Sandhills region is the largest sand dune region in the Western Hemisphere, covering 50,000 km^2^ (1). Despite the semiarid climate, more than 1,500 lakes have formed in depressions between grass-stabilized dunes (2). Most of these lakes are shallow, with only 5% exceeding 2.5 m in depth (3). The lakes vary significantly in their geochemistry, with alkalinity from 0.0 mg/liter to >90,000 mg/liter (4), pH from neutral to 10.8 (1, 5), and salinity from 200 mg/liter to >100,000 mg/liter of total dissolved solids (TDS) (5). In addition to variance between the lakes, evaporation drives seasonal geochemical changes within the lakes (6). Though these alkaline systems are well described, their microbial communities remain undescribed.

Over a 2-year period (2007 to 2008), water and sediment samples were collected from the littoral zone of saline, alkaline lakes in the Nebraska Sandhills (Table 1). Water samples (1 liter) were collected from eight lakes in sterile, plastic bottles by immersion below the lake surface and then filtered through nitrocellulose membranes (Whatman 7182-002). Filters were lyophilized and stored with desiccant. DNA was extracted using the Qiagen BioSprint 96 One-For-All vet kit (7). Sediment samples were collected from five lakes (Border, Ellsworth, Kokjohn, Merritt, and Tree Claim) by collecting ca. 25 g of sediment directly into sterile polypropylene tubes. Sediment was pelleted by centrifugation (8) and stored at –20°C until DNA extraction with the Mo Bio PowerSoil DNA isolation kit (using the manufacturer’s protocol). The V1-V2 region of the 16S rRNA gene was amplified with bacterium-specific primers and sequenced using the Roche-454 GS FLX system for all samples (7, 9). Sequence processing was completed using QIIME 1.8.0 (10). Chimeric sequences were identified with ChimeraSlayer (11), and reads of <150 bp or with a mean quality score (Q) of <25 were discarded. Fifteen samples yielded a total of 152,015 high-quality reads (230 bp mean length, 10,134 mean reads per sample). Taxonomy was assigned in reference to Greengenes v13_8 (12) with a 97% operational taxonomic unit (OTU) identity threshold.

**TABLE 1:** Summary of geochemical and sequence data by sample site^*a*^

Lake	Coordinates	pH^*b*^	Alkalinity^*b*^ (mg/liter CaCO_3_)	Conductivity^*b*^^,^^*e*^ (μS/cm)	Sample date (mo/yr)	Source material	No. of reads	Taxonomic identification (no. of reads [%])^*c*^	Total no. of reads identified as chloroplasts (%)^*d*^
Border	41.79386°N, 102.53521°W	9.8–10.3	9,300–71,400	24,500–65,945	6/2007	Water	11,587	*Proteobacteria* (6,500 [56.1]), *Cyanobacteria* (2,344 [20.2]), *Bacteroidetes* (793 [6.8])	1,725 (14.9)
					6/2008	Water	11,862	*Cyanobacteria* (6,332 [53.4]), *Proteobacteria* (2,039 [25.6]), unassigned (1,297 [10.9])	3,027 (25.5)
					10/2008	Water	12,771	*Proteobacteria* (33,916 [30.7]), *Cyanobacteria* (3,769 [29.5]), *Bacteroidetes* (2,233 [17.5])	181 (1.4)
Ellsworth	42.06078°N, 102.28409°W	9.7	2,290	13,210	10/2008	Water	10,946	*Actinobacteria* (7,174 [65.6]), *Proteobacteria* (2,048 [18.7]), unassigned (650 [5.9])	169 (1.5)
Kokjohn	41.78245°N, 102.52274°W	9.5–9.9	2,672–27,200	6,070–70,000	6/2008	Water	10,651	*Actinobacteria* (4,647 [43.6]), *Proteobacteria* (3,277 [30.8]), unassigned (1,027 [9.6])	31 (0.3)
					10/2008	Sediment	7,334	*Proteobacteria* (2,811 [38.3]), unassigned (1,729 [23.6]), *Firmicutes* (795 [10.8])	30 (0.4)
Merritt	42.06846°N, 102.29020°W	9.4	390–3,220	8,330	10/2008	Water	9,803	*Cyanobacteria* (6,630 [67.6]), *Proteobacteria* (1,721 [17.6]), *Actinobacteria* (859 [8.8])	13 (0.1)
					10/2008	Sediment	4,198	*Proteobacteria* (1,138 [27.1]), unassigned (752 [17.9]), *Bacteroidetes* (652 [15.5])	324 (7.7)
Perrin	41.76924°N, 102.51555°W	8.6–9.0	450–522	800–1,040	6/2007	Water	12,099	*Proteobacteria* (5,854 [48.4]), *Bacteroidetes* (3,391 [28.0]), *Actinobacteria* (1,725 [14.3])	184 (1.5)
Smith	41.78609°N, 102.52386°W	8.3–8.9	470–502	148–890	6/2007	Water	11,732	*Cyanobacteria* (3,659 [31.2]), *Proteobacteria* (3,500 [29.8]), *Actinobacteria* (2,090 [17.8])	115 (1.0)
					10/2008	Water	10,566	*Cyanobacteria* (4,617 [43.7]), *Proteobacteria* (2,501 [23.7]), unassigned (1,875 [17.7])	4,119 (39.0)
Tree Claim	41.78248°N, 102.49649°W	7.5–9.9	501–9,800	1,700–17,866	6/2008	Water	9,404	*Actinobacteria* (3,748 [39.9]), *Proteobacteria* (2,155 [22.9]), *Bacteroidetes* (1,078 [11.5])	79 (0.8)
					10/2008	Water	9,749	*Cyanobacteria* (6,553 [67.2]), *Proteobacteria* (2,045 [21.0]), unassigned (563 [5.8])	80 (0.8)
					10/2008	Sediment	7,016	*Cyanobacteria* (3,573 [50.9]), *Proteobacteria* (1,506 [21.5]), unassigned (815 [11.6])	2,066 (29.4)
Louden	42.07929°N, 102.20402°W	9.3	2,810	9,450	6/2008	Water	12,297	*Cyanobacteria* (10,525 [85.6]), *Proteobacteria* (784 [6.4]), unassigned (318 [2.6])	104 (0.8)

a Geochemical values are reported as a range of observed values, when possible, to account for seasonal variation.

b Values from Shaffer et al. (6), Roberts (7), Zlotnik et al. (14), and Shinneman et al. (15).

c Read numbers were calculated by multiplying the total number of reads by the percentage of reads assigned to the taxon. *Cyanobacteria* include chloroplast-identified sequences.

d Total number of reads and percentage of total reads identified as chloroplasts.

e μS, microSiemens.

The distribution of taxa varied among the lakes and seasons (Table 1). *Cyanobacteria*, *Proteobacteria*, *Actinobacteria*, and *Bacteroidetes* were the most frequently identified phyla in the water samples. In some cases, the majority of cyanobacterial reads were classified as chloroplasts (Table 1). Several sandhill lakes have abundant algal populations (13). Chloroplast sequences were therefore not removed, as they are a marker of potential eukaryotic primary productivity.

*Cyanobacteria*, *Proteobacteria*, and *Actinobacteria* were also commonly identified in sediment samples (Table 1). Sediment samples from Border and Ellsworth were excluded from downstream analysis due to low read counts (<2,000). Sequences associated with taxa capable of anoxygenic photosynthesis (*Chromatiaceae*) were identified in Kokjohn sediment, consistent with purple pigments observed during sample processing. A lack of archaeal identification in the samples is expected as a consequence of bacterium-specific primers.

These samples indicate that microbial populations vary among the alkaline lakes. More detailed analyses of aqueous and sedimentary geochemistry and hydrology across diurnal and seasonal timescales are required to discern meaningful differences in community structures.

### Data availability.

DNA sequences from this project were deposited in the NCBI Sequence Read Archive under the accession no. SRP156869.
